# A Rare Case of Bipolar Clavicle Fracture

**DOI:** 10.1155/2016/4309828

**Published:** 2016-03-09

**Authors:** Matthew A. Yalizis, Gregory A. Hoy, Eugene T. H. Ek

**Affiliations:** ^1^Melbourne Orthopaedic Group and Department of Orthopaedic Surgery, University of Melbourne, Austin Hospital, Melbourne, Australia; ^2^Sydney Shoulder and Elbow Specialists, Australia; ^3^Department of Orthopaedics, Dandenong Hospital, Monash University, Australia; ^4^Department of Surgery, Monash Medical Centre, Monash University, Melbourne, Australia

## Abstract

Segmental or bipolar fractures of the clavicle generally refer to a concomitant ipsilateral distal clavicle and midshaft clavicle fracture. These injuries are exceedingly rare and are generally secondary to higher energy injuries. We report a case of a 38-year-old male who sustained a left bipolar clavicle fracture after falling from a push bike while riding recreationally which unusually involved the medial and lateral ends of the clavicle and not the midshaft as previously reported in other patients. The patient's exact fracture configuration was not immediately apparent highlighting the need for careful examination of the whole clavicle in order to not miss a bipolar fracture.

## 1. Introduction

Clavicle fractures are common injuries and are commonly managed with operative fixation. However, bipolar or segmental injuries require a unique combination of forces to result in the injury being sustained. With higher energy injuries, there may also be damage to soft tissue surrounding structures such as the coracoclavicular ligaments, acromioclavicular joint capsule, and underlying neurovascular structures.

## 2. Case Report

A 38-year-old right hand dominant male sustained an injury to his left shoulder girdle while riding a push bike to work at moderate speed. He was seen in the emergency department of a metropolitan hospital and referred to fracture clinic for further management of his injury.

**Figure 1:**
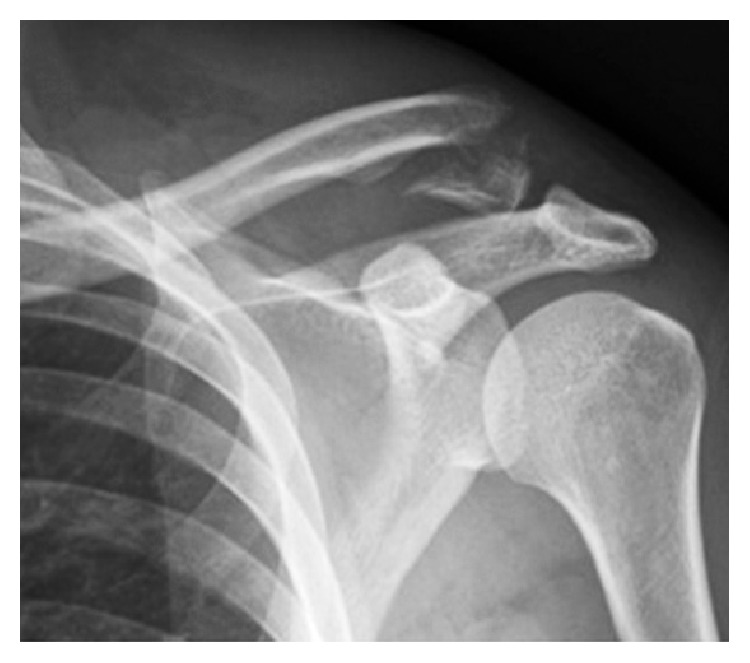
Initial radiographs prior to osteosynthesis.

He was seen in the specialist fracture clinic four days after his fracture was sustained. Examination revealed a closed injury to the left shoulder girdle and localized pain over the lateral aspect of his clavicle, with associated tenderness and crepitus. Initial radiographs revealed a displaced left distal clavicle fracture with no disruption to the coracoclavicular ligaments evident (Figure 1). Radiographs also demonstrated mildly displaced fractures of the left third and fourth ribs posteriorly. Formal radiology reporting also concurred with the above findings.

**Figure 2:**
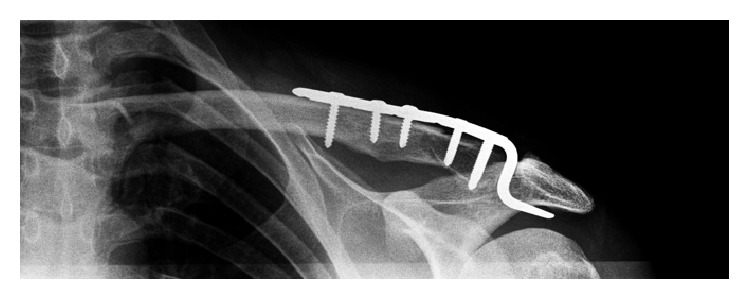
Initial hook plate fixation of distal clavicle.

The decision was made to proceed with surgical fixation of the fracture given its displaced and distal nature. He underwent operative fixation of the left distal clavicle fracture nine days after his original injury. A 6-hole hook plate was used to stabilise the fracture. Fracture reduction was confirmed on fluoroscopy and the position of the plate was also found to be in satisfactory position (Figure 2). Gentle range of motion exercises were commenced in the immediate postoperative period. His wound was checked at two weeks postoperatively and postoperative radiographs displayed his fracture alignment and hardware position to be satisfactory.

**Figure 3:**
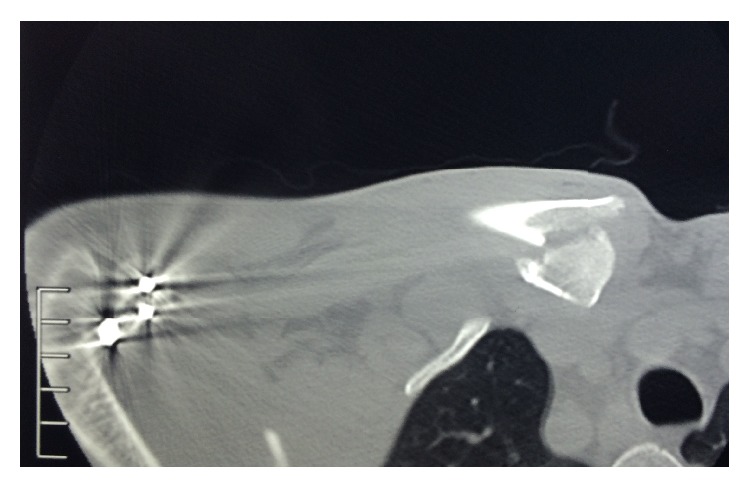
Medial clavicle fracture detected on CT scan.

Despite his uneventful recovery from the operative fixation, he had ongoing pain at the time of postoperative review more specifically over the medial clavicular region. Review of the preoperative radiographs in two views did not reveal any injury to that region. Concomitant medial clavicle pathology was suspected given his ongoing pain and hence a computerised tomography scan (CT scan) was organised after the postoperative review which was 28 days after the injury (Figure 3). The imaging revealed a comminuted intra-articular fracture of the medial end of the clavicle. This fracture was not appreciated on earlier imaging of the affected region nor on clinical examination during the initial presentation.

**Figure 4:**
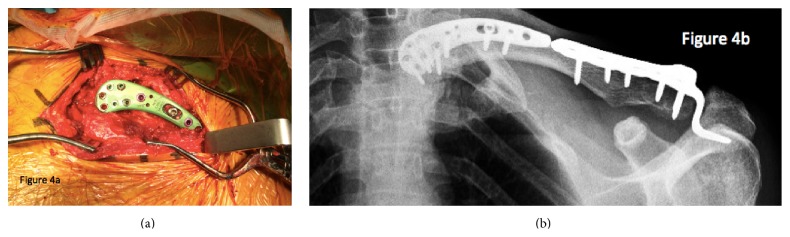
(a) Intraoperative photograph depicting the medial clavicular fixation. (b) Final fixation construct with both medial and lateral plates after healing had occurred.

He underwent operative fixation of the medial clavicle fracture on day 43 after injury where a distal clavicle plate was fashioned to fit to the medial side of the clavicle. The fracture was an oblique shear pattern and required some local bone grafting given its chronicity. The fracture was taken down and mobilised and was fixed with plate fixation. Screw fixation alone was inadequate in obtaining secure purchase given its chronicity, fracture pattern, and fragment size. Fluoroscopy was performed intraoperatively which demonstrated anatomical reduction of the fracture and satisfactory position of the hardware (Figures 4(a) and 4(b)). His hook plate was removed 3 months after the initial procedure to fix his distal clavicle and he has since that time regained a pain-free functional range of motion in his left shoulder. Decision was made to leave the medial plate in situ because it was not affecting his clinical progress and posed further surgical risk to be removed.

## 3. Discussion

Clavicle fractures are common injuries and comprise 4% of all fracture in adults [1]. Segmental clavicle fractures on the other hand are exceedingly uncommon and are very sparsely reported throughout the literature. Segmental fractures generally refer to a concomitant ipsilateral distal clavicle and midshaft clavicle fracture [2]. Bipolar fractures of the clavicle specifically refer to fractures which occur at the medial and lateral ends of the clavicle and are rarer than the segmental type [3]. Being even rarer than the segmental type clavicle fracture, they are more susceptible to being missed due to failure to look for a second injury after the initial diagnosis [1] and the rarity of the lesion itself [3]. Other bipolar clavicular injuries include the floating clavicle where there is a dislocation of both medial and lateral ends of the clavicle. The earliest report of this injury in the literature was in 1831 where its natural history was believed to be associated with relatively normal shoulder function [4]. There has also been a more recent report of a floating clavicle which was also treated nonoperatively at the patient's request and recovered a pain-free functional range of motion at 1-year follow-up [5].

Another report of a bipolar clavicular physeal injury exists in a child which was treated nonoperatively and recovered normal function [6]. However, bipolar fractures tend to occur in adults. There is a report of a bipolar clavicle fracture which was treated nonoperatively and recovered normal function [7]. These injuries are rare but can complicate clinical progress if they are missed and may lead to revision procedures. Accordingly, it is imperative to carefully assess any fractured clavicle along its whole length both clinically and radiologically and potentially utilising adjunct computerised tomography where required. Careful examination of the whole clavicle is warranted, despite diffuse shoulder girdle swelling, in order not to miss a bipolar fracture.
